# Characteristics of COVID-19 vaccinated and unvaccinated patients admitted to Careggi University Hospital, Florence, Italy

**DOI:** 10.1007/s11739-023-03231-w

**Published:** 2023-02-28

**Authors:** Riccardo Paggi, Anna Barbiero, Tommaso Manciulli, Andreea Miftode, Marta Tilli, Filippo Lagi, Jessica Mencarini, Beatrice Borchi, Marco Pozzi, Filippo Bartalesi, Michele Spinicci, Lorenzo Martini, Alessandra Coppola, Carlo Nozzoli, Adriano Peris, Manuela Bonizzoli, Filippo Pieralli, Alessandro Bartoloni, Lorenzo Zammarchi

**Affiliations:** 1 Department of Experimental and Clinical Medicine, University of Florence, Florence, Italy; 2 Infectious and Tropical Diseases Unit, Careggi University Hospital, Florence, Italy; 3 Internal Medicine Unit 2, Careggi University Hospital, Florence, Italy; 4 Internal Medicine Unit 1, Careggi University Hospital, Florence, Italy; 5 Intensive Care Unit and Regional ECMO Referral Centre, Azienda Ospedaliero Universitaria Careggi, Florence, Italy; 6 High-Intensity Internal Medicine Unit, Careggi University Hospital, Florence, Italy

**Keywords:** Covid-19, SARS-CoV-2 vaccines, COVID-19 serological testing, Hospitalisation, Breakthrough infection, Mortality

## Abstract

More than 11.5 billion COVID-19 vaccine doses have been administered around the world. Although vaccine effectiveness for severe infections is reported to be 89.0%, breakthrough infections are common and may lead to severe outcome in fragile population. We conducted a real-world observational study on 420 COVID-19 admitted patients from July 2021 to January 2022 in a tertiary level Italian hospital. We collected patient’s vaccination and SARS-CoV-2 serological status, SARS-CoV-2 treatments, oxygen supports, intensive (ICU) and subintensive (sub-ICU) care unit admissions, length of staying (LoS) and in-hospital mortality. One-hundred-seventy-two vaccinated and 248 unvaccinated patients were admitted during the study period. Vaccinated group (Vg) had a significantly more elevated Charlson Comorbidity Index than Unvaccinated group (UVg), and no statistical differences were found in terms of in-hospital mortality, LoS or ICU and sub-ICU admissions. Among Vg, anti-S antibodies were detected in 86.18% of patients (seropositives). Vaccinated seronegative patients’ in-hospital mortality was significantly higher than vaccinated seropositive patients (33.33% vs 10.69%, *p* = 0.0055): in particular, mortality rate in 45–69 years old population was higher in vaccinated seronegative group, and comparable in patients ≥ 70 years old. No differences in terms of outcome were registered between Vg and UVg, taking into account that Vg was considerably older and with more comorbidities. In line with other recent observations, higher mortality rate was evidenced for seronegative vaccinated patients. Primary prophylaxis and early treatments result to be necessary, especially for older and immunosuppressed populations.

**Supplementary Information:**

The online version contains supplementary material available at 10.1007/s11739-023-03231-w.

## Introduction

Since the beginning of the SARS-CoV-2 pandemic, several efforts have been made to develop effective vaccines against2 Coronavirus Disease 2019 (COVID-19). The case fatality rate in the pre-vaccine era was up to 40% [1]. Vaccines based on several technologies are currently available and have been broadly used.

More than 11.5 billion vaccine doses were administered worldwide, with 4.5 billion people having completed a vaccination cycle [2]. In Italy, by January 2023, more than 90% of the over 12 years old population (49 million) was vaccinated with a full dose vaccine schedule. An estimated 90%, 92%, 94%, and 95% of people ranging between 50–59, 60–69, 70–79, and over 80 years old, respectively, were inoculated with one or two doses [3].

According to Phase III trials, mRNA-based vaccines have been shown to prevent the development of severe forms of COVID-19; these percentages are lower for vaccines using adenovirus vectors [4–7]. A recent meta-analysis [8] showed that the overall vaccine effectiveness for severe infection is 89%. Despite the evident efficacy toward preventing severe disease and COVID-19 related hospitalisations, other factors such as age, comorbidities, and treatment availability may contribute to the outcome of SARS-CoV-2 vaccinated patients hospitalised for COVID-19 [9–12]: not only the elderly population seems more susceptible to severe disease due to waning immunity and breakthrough infections [13],but recent data also suggest that seronegative (anti-S IgG antibodies, Abs) vaccinated patients are at higher risk for severe breakthrough infections [12, 14]. Incidence of COVID-19 related mortality in vaccinated patients seems to increase with age, comorbidities and male sex, and appeared to be particularly correlated with some ethnicities. As well, several conditions and comorbidities such as Down’s syndrome, immune-depression, neurological disorders, pulmonary and heart diseases are likely related with higher mortality and admission rates in the vaccinated population [15]. For this reason, considering the constantly increasing vaccination coverage that is being reached in many countries, it is important that risk factors and predictors of admission and mortality keep being studied and analysed both in vaccinated and unvaccinated people.

We conducted a real-world observational study on patients admitted to our hospital for COVID-19, aiming to determine differences in demographic features, treatments, and outcomes according to vaccination status and serological status.

## Materials and methods

This is an observational, retrospective, monocentric study. We collected data from COVID-19patients admitted at Careggi University Hospital, Florence, Italy. Inclusion criteria were:

- people ≥ 18 years old;
- confirmed SARS-CoV-2 positivity by polymerase chain reaction (PCR) on a nasopharyngeal swab or bronchoalveolar lavage;
- admission at Careggi University Hospital due to COVID-19.

We excluded patients with a positive SARS-CoV-2 nasopharyngeal swab admitted for clinical conditions unrelated to COVID-19. We excluded patients with no COVID-19 related symptoms.

Data were retrieved from electronic medical records of patients admitted to infectious disease (ID) ward, internal medicine (IM) wards, subintensive care unit (sub-ICU) and intensive care unit (ICU) wards, between July 1st, 2021 and January 27th, 2022.

Collected data included: demographic features, comorbidities, SARS-CoV-2 vaccination status (including whether the patient was partly or fully vaccinated, or boosted), admission ward, COVID-19 severity scale, SARS-CoV-2 variant of concern (VOC), SARS-CoV-2 serology status, COVID-19 treatment and types of oxygen support required during the hospitalisation, organ failures occurred during the hospitalisation, admission in sub-ICU and ICU, length of hospitalisation (LoH), in-hospital mortality, discharge destination (low-care facility, home).

Data about vaccination status were confirmed by checking the regional collective prevention sanitary informative system (Sistema Informativo Sanitario di Prevenzione Collettiva). Comirnaty^®^, Spikevax^®^, Jannsen^®^ and Vaxzevria^®^ were the vaccines available during the study period.

Based on the available literature, 14 days was established as the interval necessary to build an immune response after vaccination and, therefore, consider the vaccination fully effective [16]. Consequently, we included in the vaccinated group (Vg) patients vaccinated with at least two doses of Comirnaty^®^, Spikevax^®^ or Vaxzevria^®^, with an interval of at least 14 days between the second vaccine dose and the symptom onset, or with one dose of Jannsen^®^, with an interval of at least 14 days from the dose. The unvaccinated group (UVg) includes unvaccinated patients, patients self-reported to be vaccinated by vaccines not approved in Italy (not confirmable by digital server), or patients vaccinated with only one dose or completing the vaccine schedule less than 14 days before admission. Patients with positive anti-S IgG Abs were defined as seropositive; patients with negative serology were defined as seronegative.

Regarding comorbidities, CDC defined conditions for high risk of severe COVID-19 were taken into consideration [18]. For each patient, Charlson Comorbidity Index (CCI) was calculated. COVID-19 severity was calculated according to NIH Severity Score Criteria [19].

SARS-CoV-2 VOC were analysed with Allplex™ SARS-CoV-2 Variants I Assay (Seegene).

### Outcomes

Primary outcomes were defined as LoH, sub-ICU and ICU admission and in-hospital mortality; secondary outcomes were defined as initial and overall COVID-19 severity score, usage of SARS-CoV-2 therapies and oxygen support.

The study aims to compare vaccinated and unvaccinated groups, seropositive and seronegative groups, in terms of demographics and comorbidities, admission ward, primary and secondary outcomes.

### Statistics

Descriptive analysis was employed to illustrate population characteristics. Categorical variables were evaluated with *X*^2^/Fisher's exact test. Continuous variables with Mann–Whitney test. The cumulative risk of ICU admission and in-hospital mortality was assessed using Kaplan–Meier curves. The Mantel–Haenszel method was used to produce adjusted RR for each potential confounder in turn. A multivariate analysis by Cox regression was used to examine the association between death and select variables (vaccination status, age category, Charlson comorbidity index, sex). STATA v13.0 (STATACorp, USA) was used for statistical analyses.

Three age categories were defined for Kaplan Meier analysis and multivariate analysis, dividing the population into patients < 41.5 years old, patients from 41.5 to 64.9 years old, and patients ≥ 65 years old. Last age category was chosen as representative of “aged” population [17], while the first two age categories were defined dividing in half the population between 18 and 65 years old.

## Results

During the study period, 552 patients were admitted with a positive SARS-CoV-2 nasopharyngeal swab. Of them, 420 were admitted for COVID-19. One-hundred-seventy-two patients (172/420, 41.0%) were vaccinated, 248 (248/420, 59.0%) were unvaccinated. Among vaccinated patients, 12 (7%) were younger than 41.5 years old, 28 (16.3%) were from 41.5 to 64.5, 132 (76.8%) were 65 or older. As regards unvaccinated population, 43 (17.3%) patients were younger than 41.5 year old, 125 (50.4%) were between 41.5 and 64.5 years old, 80 (32.3%) were 64.5 years old or older. General and clinical characteristics are reported in Table 1.

**Table 1:** General and clinical characteristics of COVID-19 admitted patients in a single centre in Italy from 1st July 2021 to 27th January 2022

	Vaccinated (*n* 172)	Unvaccinated (*n* 248)	*p* value
General characteristics			
Patients (*n*, %)	172 (40.95)	248 (59.05)	
Age (median **[**IQR 25–75%])	77.92 [66.17–84.30]	55.43 [44.89–69.08]	**0.001**
Age category (*n*, %)			
< 41.5 years old 55 (13.10)	12 (21.82)	43 (78.18)	
41.5–64.9 years old 153 (36.43)	28 (18.30)	125 (81.70)	
≥ 65 years old 212 (50.48)	132 (62.26)	80 (37.74)	
Male (*n*, %)	88 (51.16)	129 (52.02)	0.863
CCI (median, IQR)	5 (3–7)	3 (0–4)	**< 0.001**
Previous SARS-CoV-2 infection (*n*, %)	1 (0.58)	5 (2.02)	0.223
CDC comorbidities			
Pregnancy (*n*, %)	1 (0.58)	12 (4.84)	**0.013**
Cancer (*n*, %)	32 (18.60)	19 (7.66)	**0.001**
Diabetes (*n*, %)	38 (22.09)	26 (10.48)	**0.001**
CKD (*n*, %)	29 (16.86)	6 (2.42)	**< 0.001**
Lung disease (*n*, %)	42 (24.42)	31 (12.50)	**0.002**
Dementia (*n*, %)	39 (22.67)	20 (8.06)	**< 0.001**
Smoking (*n*, %)	63 (36.63)	67 (27.02)	**0.036**
Heart disease (*n*, %)	96 (55.81)	70 (28.23)	**< 0.001**
HIV/AIDS (*n*, %)	1 (0.59)	1 (0.40)	0.794
Immunodeficiency (*n*, %)	8 (4.65)	4 (1.61)	0.066
Overweight/obesity (*n*, %)	66 (38.37)	90 (36.29)	0.644
Haemoglobin disease (*n*, %)	6 (3.49)	2 (0.81)	**0.048**
SOT/BMT (*n*, %)	3 (1.74)	2 (0.81)	0.384
Stroke (*n*, %)	23 (13.37)	19 (7.66)	0.055
Liver disease (*n*, %)	9 (5.23)	8 (3.23)	0.305
Down syndrome (*n*, %)	1 (0.58)	0 (0.00)	0.229
Substance abuse (*n*, %)	3 (1.74)	3 (1.21)	0.650

*CCI* Charlson Comorbidity Index, *CKD* chronic kidney disease, *IQR* inter quartile range, *SD* standard deviation, *SOT/BMT* solid organ or bone marrow transplant. P-values are marked in bold when <0.05.

The vaccinated cohort was significantly older than the unvaccinated cohort (median [IQR 25–75%]: 77.92 [66.17–84.30] vs 55.43 [44.89–69.08], *p* = 0.001), with several comorbidities significantly more frequent in the first group: heart disease, lung disease, chronic kidney disease, diabetes mellitus, dementia, former or present cancer, haemoglobin disease. Charlson comorbidity index was significantly higher in the Vg (5 vs 3, *p* < 0.001).

Ninety-five percent (399/420) of the entire population was admitted in ordinary wards (ID and IM wards), 4.5% (19/420) in sub-ICU and 0.6% (2/420) in ICU, without statistically significant differences between the two cohorts (Table 2). Among the 246 sequenced variants, Delta VOC was the most common. Regarding vaccination type, data were available for 131 patients in clinical charts: the most common vaccine was Comirnaty^®^ (79.39%), followed by Vaxzevria^®^ (13.74%), Janssen^®^ (5.34%) and Spikevax^®^ (1.53%).

**Table 2:** Hospital admission characteristics of COVID-19 admitted patients in a single centre in Italy from 1st July 2021 to 27th January 2022, divided in vaccinated and unvaccinated groups

	Vaccinated (*n* 172)	Unvaccinated (*n* 248)	*p* value
Admission ward			
Ordinary ward (*n*, %)	164 (95.35)	235 (94.76)	0.903
Sub ICU (*n*, %)	7 (4.07)	12 (4.84)	
ICU (*n*, %)	1 (0.58)	1 (0.40)	
SARS-CoV-2 variants			
Alpha (*n*, %)	0 (0.00)	1 (0.42)	0.243
Delta (*n*, %)	89 (52.35)	144 (60.25)	
Omicron (*n*, %)	7 (4.12)	5 (2.09)	
Undetermined (*n*, %)	74 (43.53)	89 (37.24)	
Vaccination type			
Comirnaty (*n*, %)	104 (79.39)	–	
Spikevax (*n*, %)	2 (1.53)	–	
Vaxzevria (*n*, %)	18 (13.74)	–	
Janssen (*n*, %)	7 (5.34)	–	

Percentages are calculated per group

*ICU* Intensive Care Unit

According to the NIH severity score, 69.5% of the population was admitted with severe/critical COVID-19, with similar proportions between Vg and UVg. In the supplementary material (supplementary materials—Table 1) proportions of COVID-19 severity and organ insufficiencies during the hospitalisation are reported, with no statistically significant differences, except for incidence of renal insufficiency, which was higher in Vg (11.0 vs 3.2%, *p* = 0.001).

In Table 3 SARS-CoV-2 therapies and oxygen support administered to the two cohorts are listed. Monoclonal antibodies (mAbs) were administered to 27.8% of UVg, significantly more than the Vg (8.1%, *p* < 0.001). Moreover, UVg was significantly more prone to require anti-IL6 inhibitors administration (*p* = 0.023), as well as high flow nasal cannula (HFNC) (*p* = 0.036) and extracorporeal membrane oxygenation (ECMO, *p* = 0.040).

**Table 3:** In-hospital mortality, length of staying, admission to ICU and sub-ICU, SARS-CoV-2 treatments and oxygen support in COVID-19 admitted patients in a single centre in Italy from 1st July 2021 to 27th January 2022, divided in vaccinated and unvaccinated groups

	Vaccinated (*n* 172)	Unvaccinated (*n* 248)	*p* value
In-hospital mortality			
Overall (*n*, %)	29 (16.86)	27 (10.89)	0.068
Population < 41.5 years old (*n*, %)	0/12 (0.0)	2/43 (4.65)	1.000^1^
Population from 41.5 to 64.9 years old (*n*, %)	3/28 (10.71)	6/125 (4.80)	**0.012**^1^
Population ≥ 65 years old (*n*, %)	26/132 (19.70)	19/80 (23.75)	0.217^1^
Other primary oucomes			
LoH (mean days ± SD)	10.8 ± 8.0	11.5 ± 13.1	0.960
ICU admission	11 (6.5)	25 (10.3)	0.176
Sub ICU admission	25 (14.5)	42 (17.0)	0.960
SARS-CoV-2 treatments			
Casirivimab/Imdevimab treatment (*n*, %)	14 (8.14)	69 (27.82)	**< 0.001**
mAbs, preventive (*n*, %)	4 (2.33)	3 (1.21)	0.380
Steroids (*n*, %)	153 (88.9)	224 (90.32)	0.649
Remdesivir treatment (*n*, %)	44 (25.58)	67 (27.02)	0.743
Tocilizumab (*n*, %)	12 (6.98)	35 (14.11)	**0.023**
O2 therapy			
LFNC (*n*, %)	149 (86.63)	226 (91.13)	0.142
HFNC (*n*, %)	40 (23.26)	81 (32.66)	0.036
CPAP (*n*, %)	23 (13.37)	46 (18.55)	0.159
NIV (*n*, %)	37 (21.51)	53 (21.37)	0.972
OTI (*n*, %)	9 (5.23)	21 (8.47)	0.206
ECMO (*n*, %)	0 (0.00)	6 (2.42)	**0.040**

Percentages are calculated per group

*CPAP* continuous positive airway pressure, *ECMO* extracorporeal membrane oxygenation, *HFNC* high flow nasal cannula, *LFNC* low flow nasal cannula, *mAbs* monoclonal antibodies, *LoH* length of hospitalisation, *NIV* non invasive ventilation, *OTI* orotracheal intubation. P-values are marked in bold when <0.05.

^1^*p*-values were calculated with log-rank test

**Fig. 1:**
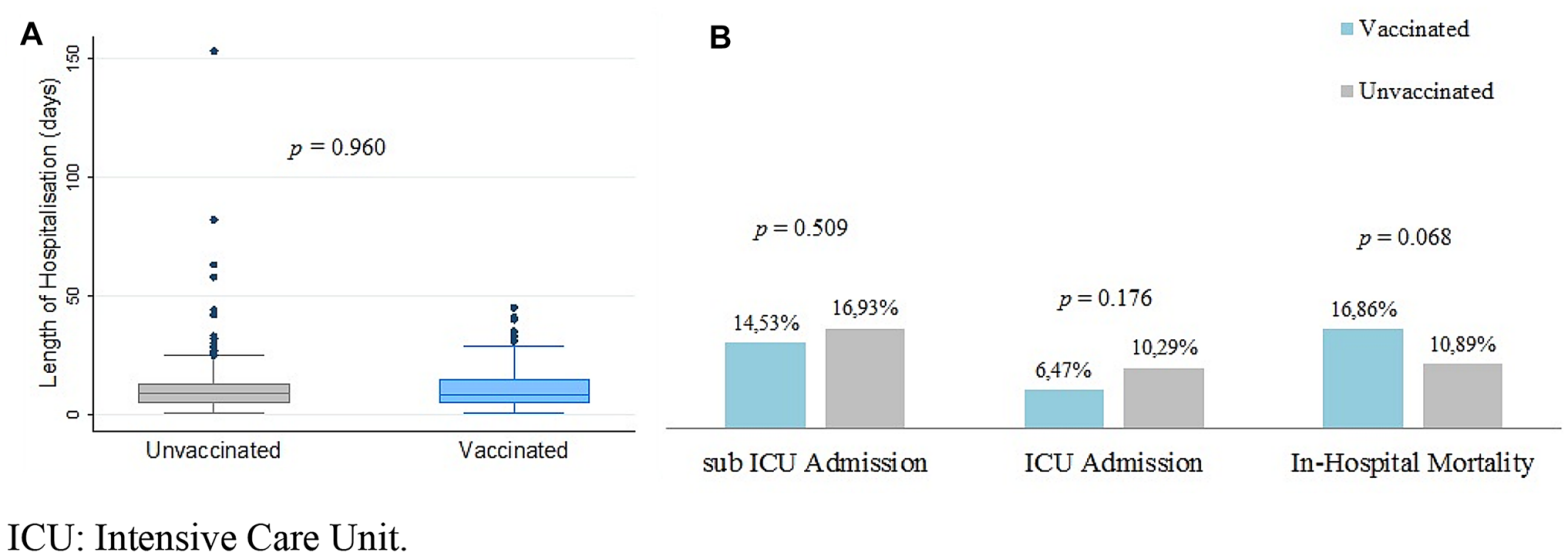
Length of hospitalisation (**a**), sub-ICU and ICU admission (**b**), in-hospital mortality (**b**) of COVID-19 admitted patients in a single centre in Italy from 1st July 2021 to 27th January 2022, comparison between vaccinated and unvaccinated groups. *ICU* Intensive Care Unit

Hospital stay was 10.8 ± 8.0 days in Vg, against 11.5 ± 13.1 days for UVg. Among Vg, patients admitted in sub-ICU and ICU were 25/172 (14.5%) and 11/170 (6.5%) respectively, against 42/248 (17.0%) and 25/243 (10.3%) of UVg. There were no significant differences between the two groups. Fifty-six patients (56/420, 13.3%) died during the hospitalisation: twenty-nine (16.9%) were vaccinated against 27 unvaccinated (10.9%), while 20 patients from Vg (11.6%) were transferred to low-care facilities, against 8 (3.2%) from UVg. In-hospital mortality difference was not statistically significant (*p* = 0.068) (Fig. 1). As showed by Kaplan–Meier survival curves (supplementary materials—Fig. 1), in-hospital mortality was significantly higher in vaccinated group considering patients from 41.5 to 65 years older (log-rank test, *p* = 0.012), but vaccinated CCI was significantly higher than unvaccinated CCI in the same age category (3.39 vs 2.18, *p* = 0.021). Results are resumed in Table 3.

By Cox regression multivariate analysis, CCI was the only factor significantly associated with mortality (HR 1.28, 95% CI 1.14–1.44), while the other considered variables did not show a significant association with our outcome (supplementary materials—Table 2). In particular, vaccination was not significantly associated with mortality (HR 0.55, 95% CI 0.29–1.05).

Anti-S IgG samples were available from 152 vaccinated patients and 217 unvaccinated patients. Among Vg, 131/152 (86.2%) resulted positive, while 86/217 (39.6%) were seropositive in the UVg, being this difference statistically significant (*p* < 0.001).

The proportion of patients with primary or secondary immunosuppression was significantly higher in vaccinated seronegative (3/21) than in vaccinated seropositive (3/131) (14.3% vs 2.3%, *p* = 0.009).

**Fig. 2:**
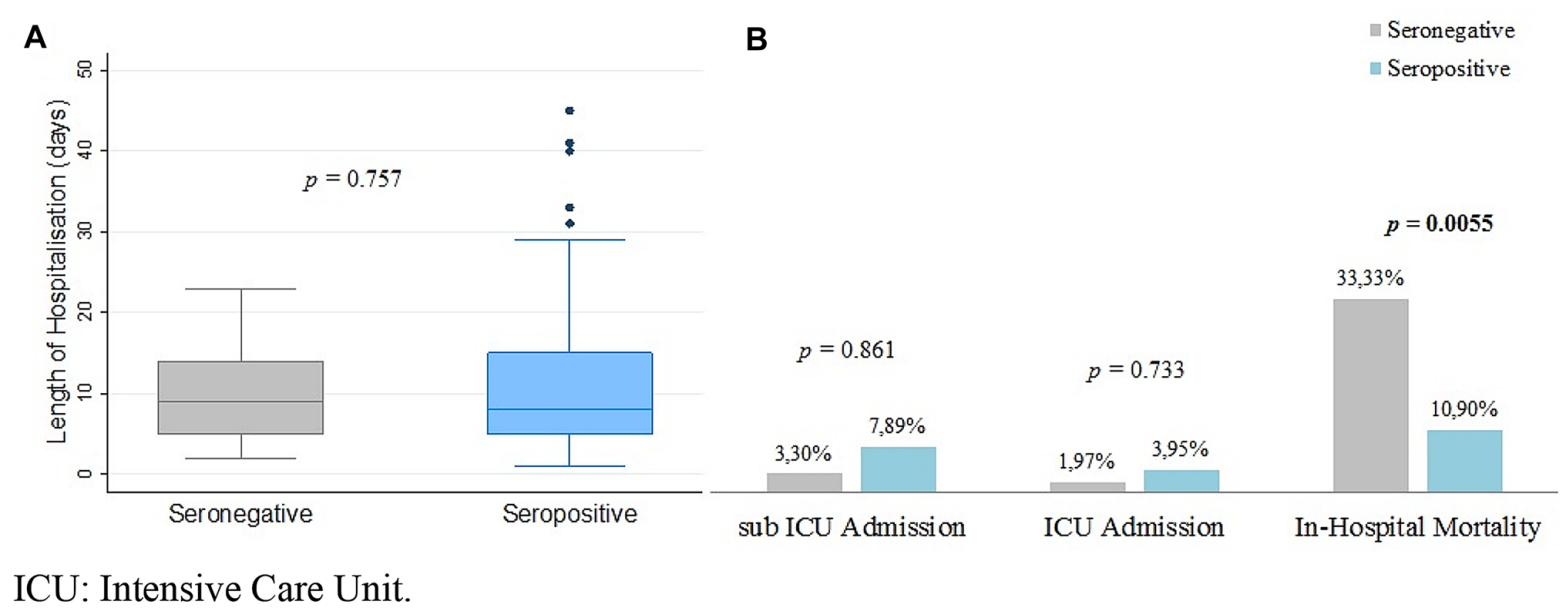
Length of hospitalisation (**a**), sub-ICU and ICU admission (**b**), in-hospital mortality (**b**) of COVID-19 admitted patients in in a single centre in Italy from 1st July 2021 to 27th January 2022 comparison between vaccinated seropositive and vaccinated seronegative groups. *ICU* Intensive Care Unit

**Fig. 3:**
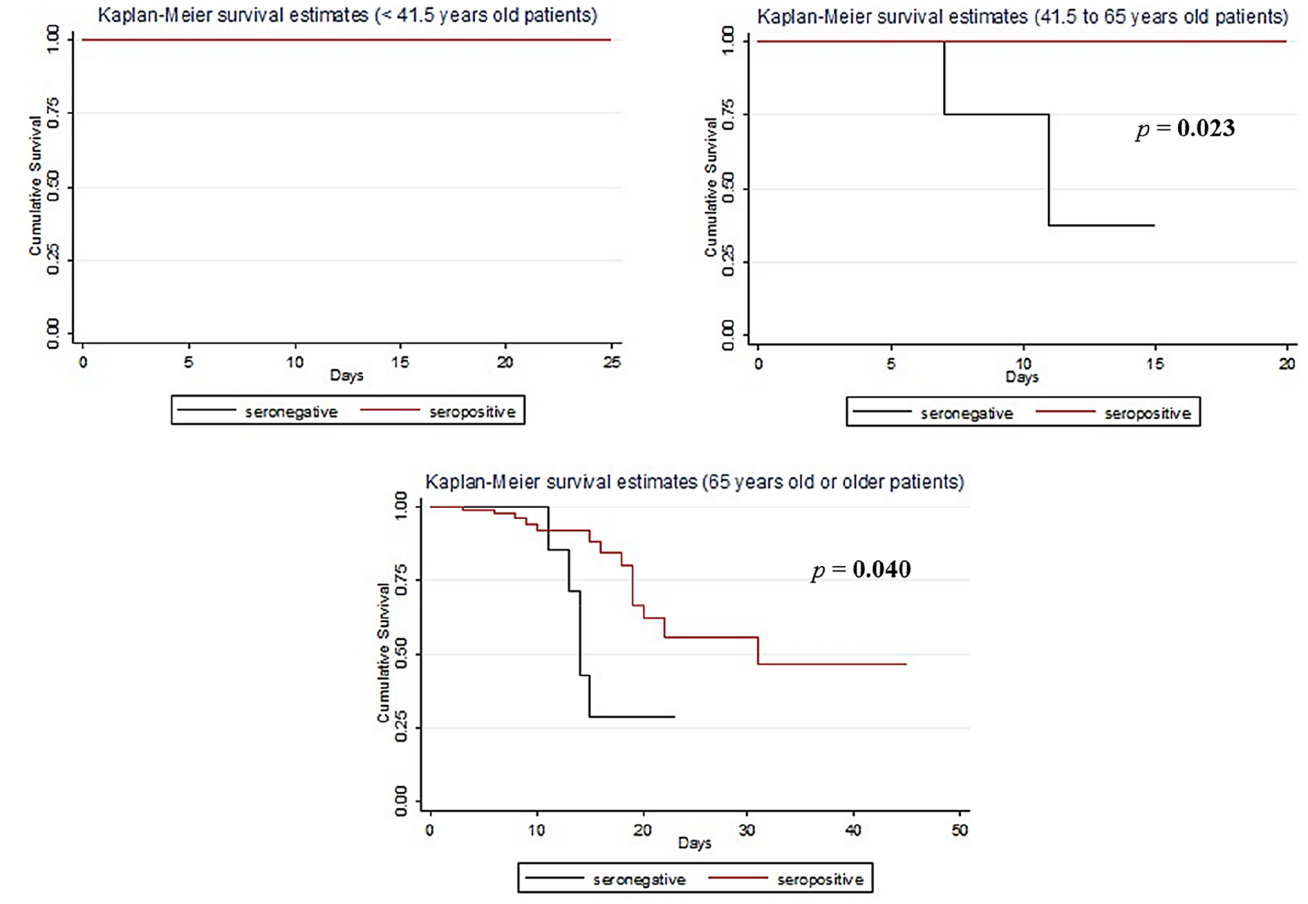
Cumulative survival of COVID-19 admitted patients vaccinated for SARS-CoV-2 in a single centre in Italy during the hospital staying, divided in age categories (< 41.5 years old, 41.5–64.9 years old, ≥ 65 years old), comparison between seropositive (anti-S IgG positive) and seronegative (anti-S IgG negative)

As exposed by Fig. 2, no significant differences resulted in terms of LoH nor ICU and sub-ICU admissions between seropositive and seronegative vaccinated groups. Seven out of 21 (33.3%) seronegative vaccinated patients died during the hospitalisation, against 14/131 (10.7%) seropositive vaccinated patients: the difference was statistically significant (*p* = 0.005), with a risk ratio of 0.298. In-hospital mortality is significantly lower in patients from 41.5 to 64.9 years old (log-rank test, *p* = 0.023) and patients ≥ 65 years old (log-rank test, *p* = 0.040), as showed in Fig. 3. No differences in terms of CCI were evidenced in the aforementioned age categories. Population characteristics and in-hospital mortality are resumed in supplementary materials—Table 3.

Differences in primary outcomes were not found in unvaccinated seropositive and seronegative subgroups (not showed).

## Discussion

Risk for COVID-19 associated mortality is notably increased by ageing, disabilities, and underlying medical conditions [20]. In our study the mortality risk among patients hospitalized for COVID-19 was 13.3%, similar to the percentage (15.1%) reported in a recent paper for “Delta VOC wave” of the pandemic [20].Vaccinated population was significantly older and with more comorbidities in respect to the unvaccinated cohort; in a fully vaccinated population, the geriatric patient is the most vulnerable, and at the highest risk for hospitalization [21, 22].

In our study, no differences in LoH, frequency of ICU and sub-ICU admission, and in-hospital mortality were evidenced between the two populations: decreased vaccine effectiveness and waning immunity in the older population [11], along with demographic and pathological characteristics of the two groups, could explain the lack of significant differences between the compared cohorts [20, 22, 23]. In our centre, in-hospital mortality of vaccinated patients between 41.5 and 64.9 years old was significantly higher than the unvaccinated patients in the same age category: interestingly, CCI was significantly higher in Vg in this age group. This observation is in line with an extensive demographic study conducted on the Scottish population, in which fully vaccinated people with more than five comorbidities were at higher risk of COVID-19-related death [10], similarly to other Italian observations [24]. As underlined in an extensive Norway register-based cohort study, in-hospital risk of death among fully vaccinated and unvaccinated patients tends to be similar [21], highlighting that factors other than vaccination need to be considered in terms of outcomes. Importantly, in our study CCI was the only factor (considering female sex, vaccination status, CCI and age categories) significantly associated with in-hospital mortality (Hazard Ratio 1.28, *p* < 0.001), leading to the conclusion that factors as comorbidities and age can importantly affect in-hospital outcome.

Comparing anti-S IgG positivity, a significant difference in in-hospital mortality emerged between vaccinated seropositive and seronegative subgroups (10% vs 33%). Notably, no in-hospital deaths were registered in the seropositive vaccinated population between 41.5 and 64.9 years old (Fig. 3), contrasting with seronegative vaccinated patients in the same age category. No vaccinated person ≤ 44 years old died during the observation comparing the two subgroups, while most of the mortality was registered in the ≥ 65 years old population, with increased in-hospital mortality in seronegative group. Notably, no significant differences in terms of CCI were evidenced in seropositive and seronegative patients of the same age category.

Of note, 14.3% of vaccinated seronegative patients were affected by primary or secondary immunosuppression, in contrast with 2.3% of vaccinated seropositive patients. This is in line with described observations of an increased ratio of breakthrough infections in vaccinated immunosuppressed patients [12, 25], although in the study of Kim et al*.* differences were not significant at multivariate analyses [12].

Correlation between level of anti-S IgG Abs and vaccine efficacy, as well as the correlation between anti-S IgG Abs and anti RBD (Receptor Binding Domain) Abs, with protective effect against symptomatic COVID-19 [26] and reinfections [14, 27] are well described in literature. Unfortunately, different laboratory methods were used for Abs level testing during our study period, leading to non-homogeneous results and different quantitative cut-off determining positivity. Moreover, we tested only anti-S IgG Abs, since our laboratory does not determine neutralising antibodies titre for clinical purpose nor cellular immunity effect [28].

In our study, some significant differences emerged between anti-SARS-CoV-2 therapies and oxygen support therapies. Monoclonal antibodies (casirivimab-imdevimab) were significantly more used in the UVg, due to the higher proportion of seronegative patients (seronegative status is a required criterion for the treatment administration). Tocilizumab and HFNC also were significantly more used in the same population as a proxy for a more severe development of the illness in the UVg.

These findings suggest that unvaccinated patients’ hospital care costs are higher than in the Vg; differences in costs could be even higher if we consider the high number of vaccine-preventable hospitalisations [29, 30].

## Strengths and limitations

Several limitations of this study can be evidenced. First of all, the study is a monocentric retrospective study. Secondly, vaccinated and unvaccinated populations were substantially different in terms of age and comorbidities, therefore difficultly comparable. Clinical behaviour following hospital guidelines and common considerations regarding vaccination status could have influenced therapeutic choices. Moreover, we included in UVg patients vaccinated with only one dose of vaccine (for vaccines requiring almost 2 doses): since the protection to the infection is proved to be present also with one dose [16], this could be interpreted as a confounding factor, improving outcomes of UVg in this study. We also considered in the UVg patients vaccinated abroad with vaccine not included in the present study analysis.

Furthermore, data collected about treatments were limited to COVID-19 antivirals and oxygen support. As such, we did not collect data on anti-coagulation therapies. However, as per our internal protocol, during the study period all COVID-19 patients routinely received prophylactic or intermediate anti-coagulation, according to the Sepsis Induced Coagulopathy (SIC) SCORE < 4 or ≥ 4, respectively, unless they had underlying condition requiring full anti-coagulation (e.g. deep vein thrombosis, pulmonary embolism, etc..). Finally we did not included data about SARS-CoV-2 VOC in the analysis. However, it should be considered that the study period (Jul 21–Jan 22) mostly reflects the period of circulation of Delta variant in our area, thus preventing any meaningful comparison between Delta and Omicron outcome [31].

On the other hand, our study offers a real-life prospective of variables and outcomes of COVID-19 hospitalised population over a 6 months observational period. Considering that most studies of vaccine efficacy are addressed to general population taking into account hospitalisation as an outcome itself, we described more specifically characteristics of vaccinated and unvaccinated population when hospitalisation occurred.

## Conclusions

In summary, considering our study outcomes, we must take in consideration that this study analysed only hospitalised patients, which are not representative of the general population: hospitalised Vg reflects the fragile part of the vaccinated general population, which is more likely to need high level care and at more risk of death. Considering the general population, vaccination prevented an important number of ICU admissions and in-hospital mortality events: although at higher risk of death, an estimated 79% of total deaths in the over 80 years old population has been prevented, as a recent Italian observation stated [29]. Since elderly population is generally considered at higher risk of in-hospital mortality [20], the lack of significance in terms of primary outcomes could be interpreted as a direct effect of the vaccine, in a population in which we would have expected important mortality rates.

Moreover, this study evidenced that primary and secondary prophylaxis measures need to be implemented in the national sanitary system. Importance of facial masks and hand hygiene is continuously underlined and proved [32–34], and pre-exposure prophylaxis in immunocompromised people with monoclonal antibody combination tixagevimab-cilgavimab is another important step for primary prophylaxis [35–37]. Early treatments in the older population represent a fundamental protective strategy as well, implementing the use of intravenous and intramuscular monoclonal antibodies [38, 39] and oral antivirals, such as molnupiravir [40] and nirmatrelvir-ritonavir [41].

In conclusion, although vaccination showed a protective effect, more evident in the seropositive population, our study underlined that other primary and secondary prophylaxis measures still have a fundamental role, particularly in at-risk population.

## Supplementary Information

Below is the link to the electronic supplementary material.

Supplementary file1 (DOCX 388 KB)

## Data Availability

The datasets generated during and/or analysed during the current study are available from the corresponding author on reasonable request.

## References

[1] Wiersinga WJ, Rhodes A, Cheng AC (2020). Pathophysiology, transmission, diagnosis, and treatment of coronavirus disease 2019 (COVID-19): a review. JAMA.

[2] WHO Coronavirus (COVID-19) Dashboard. https://covid19.who.int. Accessed 17 Jun 2022

[3] Governo Italiano—Report Vaccini Anti Covid-19. https://www.governo.it/it/cscovid19/report-vaccini/. Accessed 17 Jun 2022

[4] Polack FP, Thomas SJ, Kitchin N (2020). Safety and efficacy of the BNT162b2 mRNA Covid-19 vaccine. N Engl J Med.

[5] Baden LR, El Sahly HM, Essink B (2021). Efficacy and safety of the mRNA-1273 SARS-CoV-2 Vaccine. N Engl J Med.

[6] Sadoff J, Gray G, Vandebosch A (2021). Safety and efficacy of single-dose Ad26.COV2.S vaccine against Covid-19. N Engl J Med.

[7] Voysey M, Clemens SAC, Madhi SA (2021). Safety and efficacy of the ChAdOx1 nCoV-19 vaccine (AZD1222) against SARS-CoV-2: an interim analysis of four randomised controlled trials in Brazil, South Africa, and the UK. Lancet.

[8] Zheng C, Shao W, Chen X (2022). Real-world effectiveness of COVID-19 vaccines: a literature review and meta-analysis. Int J Infect Dis.

[9] Feikin DR, Higdon MM, Abu-Raddad LJ (2022). Duration of effectiveness of vaccines against SARS-CoV-2 infection and COVID-19 disease: results of a systematic review and meta-regression. Lancet.

[10] Grange Z, Buelo A, Sullivan C (2021). Characteristics and risk of COVID-19-related death in fully vaccinated people in Scotland. Lancet.

[11] Zeng Q, Yang X, Gao Q, et al (2022) Interpretation of non-responders to SARS-CoV-2 vaccines using WHO International Standard. Allergy Immunol

[12] Kim AHJ, Sparks JA (2022). Immunosuppression and SARS-CoV-2 breakthrough infections. Lancet Rheumatol.

[13] Juthani PV, Gupta A, Borges KA (2021). Hospitalisation among vaccine breakthrough COVID-19 infections. Lancet Infect Dis.

[14] Ahmed S, Mehta P, Paul A (2022). Postvaccination antibody titres predict protection against COVID-19 in patients with autoimmune diseases: survival analysis in a prospective cohort. Ann Rheum Dis.

[15] Hippisley-Cox J, Coupland CA, Mehta N (2021). Risk prediction of covid-19 related death and hospital admission in adults after covid-19 vaccination: national prospective cohort study. BMJ.

[16] Lopez Bernal J, Andrews N, Gower C (2021). Effectiveness of the Pfizer-BioNTech and Oxford-AstraZeneca vaccines on covid-19 related symptoms, hospital admissions, and mortality in older adults in England: test negative case-control study. BMJ.

[17] Geifman N, Cohen R, Rubin E (2013). Redefining meaningful age groups in the context of disease. Age.

[18] CDC (2022) People with Certain Medical Conditions. In: Cent. Dis. Control Prev. https://www.cdc.gov/coronavirus/2019-ncov/need-extra-precautions/people-with-medical-conditions.html. Accessed 20 Jul 2022

[19] Information on COVID-19 Treatment, Prevention and Research. In: COVID-19 Treat. Guidel. https://www.covid19treatmentguidelines.nih.gov/. Accessed 17 Jun 2022

[20] Adjei S (2022) Mortality risk among patients hospitalized primarily for COVID-19 during the omicron and delta variant pandemic periods—United States, April 2020–June 2022. MMWR Morb Mortal Wkly Rep. 10.15585/mmwr.mm7137a4 10.15585/mmwr.mm7137a4 PMC9484808 36107788

[21] Whittaker R, Bråthen Kristofferson A, Valcarcel Salamanca B (2022). Length of hospital stay and risk of intensive care admission and in-hospital death among COVID-19 patients in Norway: a register-based cohort study comparing patients fully vaccinated with an mRNA vaccine to unvaccinated patients. Clin Microbiol Infect.

[22] Bahl A, Johnson S, Maine G et al (2021) Vaccination reduces need for emergency care in breakthrough COVID-19 infections: a multicenter cohort study. 10 10.1016/j.lana.2021.100065 PMC8428472 34522911

[23] Fournier P-E, Houhamdi L, Colson P (2022). SARS-CoV-2 vaccination and protection against clinical disease: a retrospective study, Bouches-du-Rhône District, Southern France, 2021. Front Microbiol.

[24] Rovida F, Esposito GL, Rissone M (2022). Characteristics and outcomes of vaccinated and nonvaccinated patients hospitalized in a single Italian hub for COVID-19 during the Delta and Omicron waves in Northern Italy. Int J Infect Dis IJID Off Publ Int Soc Infect Dis.

[25] Sun J, Zheng Q, Madhira V (2022). Association between immune dysfunction and COVID-19 breakthrough infection after SARS-CoV-2 vaccination in the US. JAMA Intern Med.

[26] Feng S, Phillips DJ, White T (2021). Correlates of protection against symptomatic and asymptomatic SARS-CoV-2 infection. Nat Med.

[27] Lumley SF, O’Donnell D, Stoesser NE (2021). Antibody status and incidence of SARS-CoV-2 infection in health care workers. N Engl J Med.

[28] Jeyanathan M, Afkhami S, Smaill F (2020). Immunological considerations for COVID-19 vaccine strategies. Nat Rev Immunol.

[29] Sacco C, Mateo-Urdiales A, Rota MC Comunicato Stampa N°29/2022—Infezioni da SARS-CoV-2, ricoveri e decessi associati a COVID-19 direttamente evitati dalla vaccinazione. In: ISS. https://iss.it/-/asset_publisher/fjTKmjJgSgdK/content/id/6929701. Accessed 18 Jun 2022

[30] Du Z, Wang L, Pandey A (2022). Modeling comparative cost-effectiveness of SARS-CoV-2 vaccine dose fractionation in India. Nat Med.

[31] Monitoraggio delle varianti del virus SARS-CoV-2 di interesse in sanità pubblica in Italia. In: EpiCentro. https://www.epicentro.iss.it/coronavirus/sars-cov-2-monitoraggio-varianti-rapporti-periodici. Accessed 24 Jan 2023

[32] Li Y, Liang M, Gao L (2021). Face masks to prevent transmission of COVID-19: a systematic review and meta-analysis. Am J Infect Control.

[33] COVID-19 infection prevention and control living guideline: mask use in community settings, 22 December 2021. https://www.who.int/publications-detail-redirect/WHO-2019-nCoV-IPC_masks-2021.1. Accessed 18 Jun 2022

[34] Hirose R, Ikegaya H, Naito Y (2021). Survival of severe acute respiratory syndrome coronavirus 2 (SARS-CoV-2) and influenza virus on human skin: importance of hand hygiene in coronavirus disease 2019 (COVID-19). Clin Infect Dis Off Publ Infect Dis Soc Am.

[35] Levin MJ, Ustianowski A, De Wit S (2022). Intramuscular AZD7442 (tixagevimab–cilgavimab) for prevention of Covid-19. N Engl J Med.

[36] Wise J (2022). Covid-19: Evusheld is approved in UK for prophylaxis in immunocompromised people. BMJ.

[37] Liew J, Gianfrancesco M, Harrison C (2022). SARS-CoV-2 breakthrough infections among vaccinated individuals with rheumatic disease: results from the COVID-19 Global Rheumatology Alliance provider registry. RMD Open.

[38] Hwang Y-C, Lu R-M, Su S-C (2022). Monoclonal antibodies for COVID-19 therapy and SARS-CoV-2 detection. J Biomed Sci.

[39] Montgomery H, Hobbs FDR, Padilla F (2022). Efficacy and safety of intramuscular administration of tixagevimab–cilgavimab for early outpatient treatment of COVID-19 (TACKLE): a phase 3, randomised, double-blind, placebo-controlled trial. Lancet Respir Med.

[40] Jayk Bernal A, Gomes da Silva MM, Musungaie DB (2022). Molnupiravir for oral treatment of Covid-19 in nonhospitalized patients. N Engl J Med.

[41] Macchiagodena M, Pagliai M, Procacci P (2022). Characterization of the non-covalent interaction between the PF-07321332 inhibitor and the SARS-CoV-2 main protease. J Mol Graph Model.

