# Supplementary material for: Characteristics of COVID-19 vaccinated and unvaccinated patients admitted to Careggi University Hospital, Florence, Italy
Source: Intern Emerg Med. 2023 Feb 28;18(3):821–30. doi: 10.1007/s11739-023-03231-w (PMC9972322; doi:10.1007/s11739-023-03231-w)

**Supplementary materials - Table 1.** Clinical characteristics of COVID-19 admitted patients in a single centre in Italy from 1^st^ July 2021 to 27^th^ January 2022. Percentages are calculated per group.

|  | Vaccinated (n 172) | Unvaccinated (n 248) | *p* - value |
| --- | --- | --- | --- |
| NIH severity score  (entrance) | | | |
| Mild (n, %) | 4 (2.33) | 12 (4.84) | *p* = 0.301 |
| Moderate (n, %) | 52 (30.23) | 60 (4.19) |  |
| Severe (n, %) | 105 (61.05) | 163 (65.73) |  |
| Critical (n, %) | 11 (6.40) | 13 (5.24) |  |
| NIH severity score  (overall) | | | |
| Mild (n, %) | 4 (2.33) | 8 (3.23) | *p* = 0.736 |
| Moderate (n, %) | 32 (18.60) | 37 (14.92) |  |
| Severe (n, %) | 92 (53.49) | 135 (54.44) |  |
| Critical (n, %) | 44 (25.58) | 68 (27.42) |  |
| Organ insufficiencies | | | |
| Respiratory (n, %) | 135 (78.49) | 192 (77.42) | *p* = 0.795 |
| CV (n, %) | 22 (12.79) | 21 (8.47) | *p* = 0.151 |
| Renal (n, %) | 19 (11.04) | 8 (3.23) | ***p* = 0.001** |
| Hepatic (n, %) | 4 (2.33) | 2 (0.81) | *p* = 0.199 |
| Cerebral (n, %) | 5 (2.91) | 4 (1.61) | *p* = 0.368 |

CV: cardiovascular.

**Supplementary materials - Table 2.** Multivariate analysis by Cox regression used to examine the association between in-hospital death and selected variables (vaccination status, age category, charlson comorbidity index, sex)

|  | HR | Z | *p* value | [95% CI] |
| --- | --- | --- | --- | --- |
| Vaccination status | 0.55 | -1.80 | 0.071 | 0.29 – 1.05 |
| CCI | 1.28 | 4.22 | **<0.001** | 1.14 - 1.44 |
| Age categories | 2.03 | 1.96 | 0.050 | 1.00 – 4.10 |
| Female sex | 0.97 | -0.09 | 0.925 | 0.57 – 1.67 |

CCI: Charlson Comorbidity Index; CI: confidence interval; HR: hazard ratio.

**Supplementary materials - Table 3.** General and clinical characteristics and in-hospital mortality of SARS-CoV-2 vaccinated population in a single centre in Italy from 1^st^ July 2021 to 27^th^ January 2022, divided in seropositives (anti-S IgG positive) and seronegative (anti-S IgG negative).

.

|  | Seropositive (n 131) | Seronegative (n 21) | *p* - value |
| --- | --- | --- | --- |
| General characteristics | | | |
| Age (median  [IQR 25-75%]) | 78.17  [66.41 – 84.06] | 68.71  [59.18 – 82.86] | *p* = 0.156 |
| Age category (n, %) |  |  |  |
| < 41.5 years old 55 (13.10) | 9 (6.87) | 2 (9.52) | **-** |
| 41.5 – 64.9 years old 153 (36.43) | 19 (14.50) | 7 (33.33) | **-** |
| ≥ 65 years old 212 (50.48) | 103 (78.63) | 12 (57.14) | **-** |
| Male (n, %) | 70 (53.44) | 13 (61.09) | *p* = 0.469 |
| CCI (median, IQR) | 5 (3-7) | 6 (3-7) | *p* = 0.591 |
| In-hospital mortality | | | |
| Overall (n, %) | 14 (10.69) | 7 (33.33) | ***p* = 0.005** |
| Population < 41.5 years old (n, %) | 0/2 (0.0) | 0/9 (0.0) | - |
| Population from 41.5 to 64.9 years old  (n, %) | 0/19 (0.0) | 2/7 (28.57) | ***p* = 0.023**^1^ |
| Population ≥ 65 years old (n, %) | 14/103 (13.59) | 5/12 (41.67) | ***p* = 0.040**^1^ |

^1^ *p*-values were calculated with log-rank test

CCI: Charlson Comorbidity Index; IQR: inter quartile range; SD: standard deviation.

**Supplementary materials – Figure 1**. Cumulative survival of COVID-19 admitted patients in a single centre in Italy during the hospital staying, divided in age categories (< 41.5 years old, 41.5-64.9 years old, ≥ 65 years old), comparison between vaccinated and unvaccinated groups.


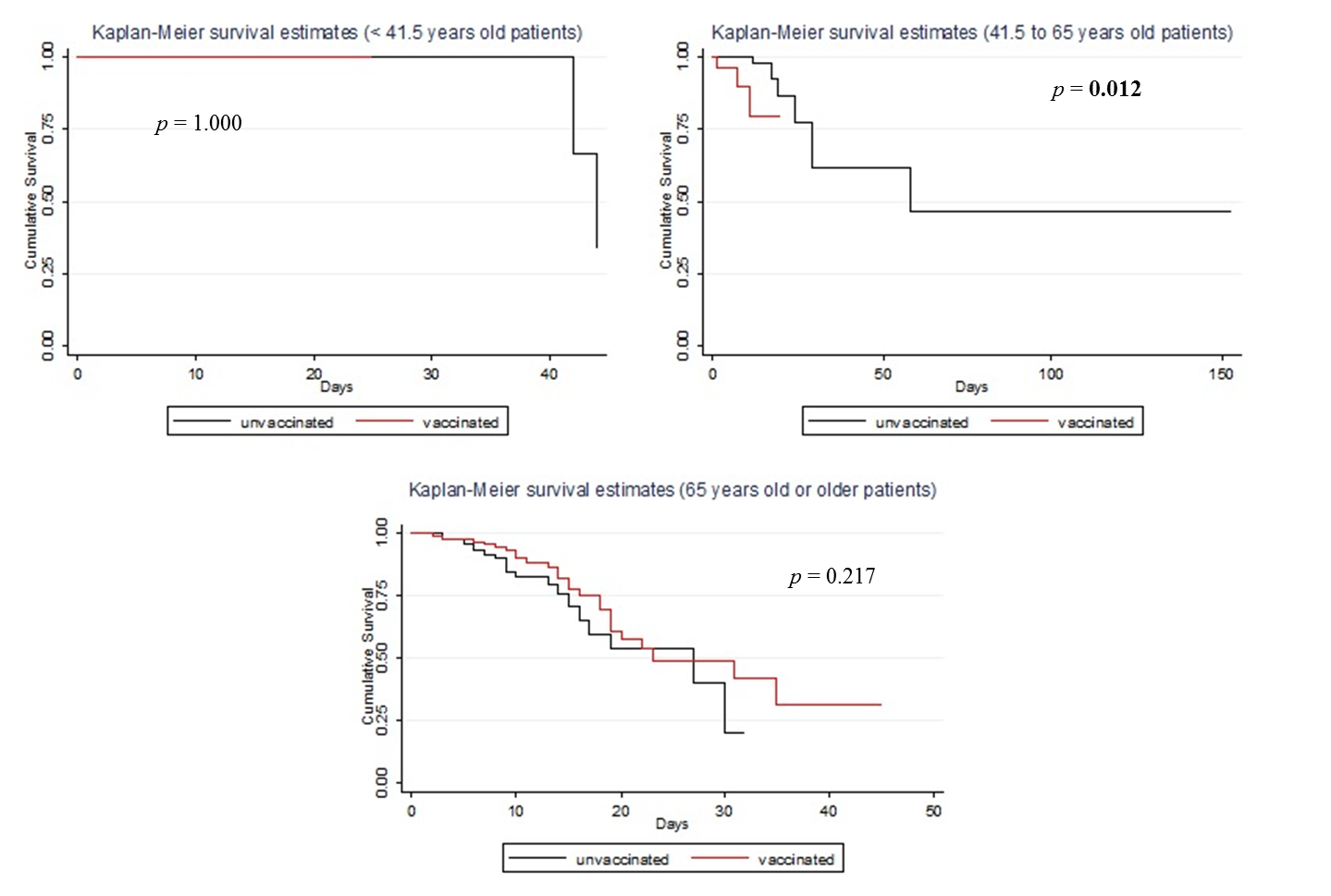

Supplement: Supplementary file 1 — Supplementary file1 (DOCX 388 KB) [file 11739_2023_3231_MOESM1_ESM.docx]
